# Force Prediction for Incremental Forming of Polymer Sheets

**DOI:** 10.3390/ma11091597

**Published:** 2018-09-03

**Authors:** Gustavo Medina-Sanchez, Alberto Garcia-Collado, Diego Carou, Rubén Dorado-Vicente

**Affiliations:** Department of Mechanical and Mining Engineering, University of Jaén, EPS de Jaén, Campus LasLagunillas, 23071 Jaén, Spain; gmedina@ujaen.es (G.M.-S.); acollado@ujaen.es (A.G.-C.); rdorado@ujaen.es (R.D.-V.)

**Keywords:** incremental forming, FEM, force prediction, numerical model, semi-analytical model, specific energy

## Abstract

Incremental sheet forming (ISF) is gaining attention as a low cost prototyping and small batch production solution to obtain 3D components. In ISF, the forming force is key to define an adequate setup, avoiding damage and reducing wear, as well as to determine the energy consumption and the final shape of the part. Although there are several analytical, experimental and numerical approaches to estimate the axial forming force for metal sheets, further efforts must be done to extend the study to polymers. This work presents two procedures for predicting axial force in Single Point Incremental Forming (SPIF) of polymer sheets. Particularly, a numerical model based on the Finite Element Model (FEM), which considers a hyperelastic-plastic constitutive equation, and a simple semi-analytical model that extends the known specific energy concept used in machining. A set of experimental tests was used to validate the numerical model, and to determine the specific energy for two polymer sheets of polycarbonate (PC) and polyvinyl chloride (PVC). The approaches provide results in good agreement with additional real examples. Moreover, the numerical model is useful for accurately predicting temperature and thickness.

## 1. Introduction

Sheet forming by means of local deformations or incremental sheet forming (ISF) is a prototyping and small batch production solution to obtain 3D components developed in the late 20th [1]. During ISF, a tool follows a 3D path deforming a flat sheet clamped to a rigid frame. This technique is noted for their low cost and good forming capabilities, beyond the forming limit curve, compared to conventional forming processes [2]. The process has applications in automotive industry [3], aeronautical industry [4] and biomedicine [5].

**Figure 1:**
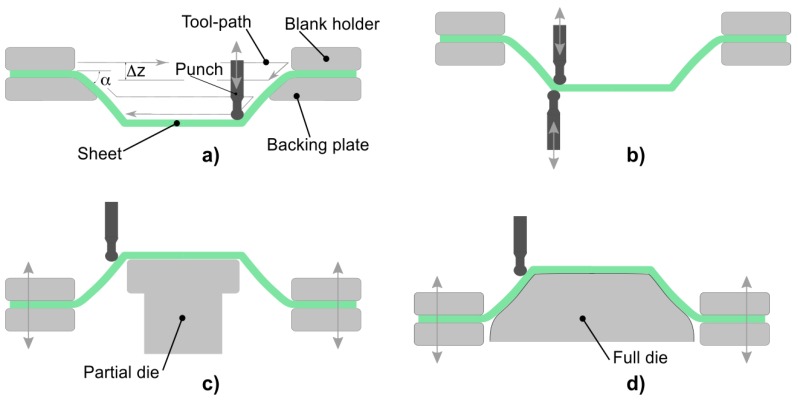
Main incremental sheet forming (ISF) techniques. (**a**) Single point incremental forming; (**b**) Two point incremental forming; (**c**) Double-sided incremental forming with partial die; (**d**) Double-sided incremental forming with full die.

The main ISF solutions are punctual incremental forming and doubled sided incremental forming assisted by a partial or full die (Figure 1). Single Point Incremental Forming (SPIF) is flexible, easy to implement low cost solution, and has been in the research spotlight in the last years. However, this process induces complex deformation mechanisms (shear, stretch and bending), which cause poor dimensional results [6], and limit its use in industrial applications [7].

SPIF is usually accomplished by a conventional Computer Numerical Control (CNC) machine equipped with a clamping frame and a punch that follows a G-code program of instructions. The deformation mechanism is related to the forming forces and, therefore, a good knowledge about forming forces allows tackling several key SPIF problems: How to improve dimensional accuracy; how to avoid machine damage and wear; how to extend the technique to different materials, etc.

Despite the industrial interest of low cost small batch production of polymer parts at room temperature [8], the extension of SPIF to these materials is currently a challenge. In addition to the SPIF drawbacks mentioned above, it is difficult to model the deformation behavior of polymer materials. Most research efforts are focused on developing prediction models for metals of known mechanical properties. When coming to glassy polymers, they suffer relaxation after yielding and, therefore, they have a complex constitutive equation with strain rate and temperature material dependence.

The study of the forming force can be an adequate strategy to advance in forming of polymer sheets. Thus, progress can be made from the extension of forming force approaches for metal sheets to polymer sheets or by developing new forming force models. To the best knowledge of the authors, existing analytical and experimental forming force models were specifically developed for metals, and the scarce numerical models for polymers are not comparable to those for metals.

Regarding the force approaches for metals, analytical models define relations between process parameters, material mechanical properties and force. For example, Bansal et al. [9] relates the forming forces with thickness, meridional and circumferential stresses, contact surface and thickness. Besides experimental models, such as the regression equation as defined by Aerens et al. [10], estimate the forming force by means of experimental data. When considering numerical simulations, they are accurate for simple geometries but computationally expensive because of the nonlinearities produced by contact area changes. Behera et al. [11] shows an extensive classification of numerical simulations works focused on metal sheets.

The existing studies about incremental forming of different polymeric materials are limited and force prediction is not their main goal. In this regard, experimental works check new applications and asses how process parameters influence response variables such as force, roughness and incremental depth [12]. On the other hand, analytical and numerical approaches try to determine deformation mechanics and failure modes [13]. Concerning these solutions, it is worth mention the theoretical model based on membrane analysis developed by Silva et al. [14], the constitutive equation, based on overstress proposed by Alkas et al. [15,16], to model viscoplastic materials with only seven material parameters, and the numerical study of Nguyen et al. [17], who considers a viscoelastic material to estimate sheet thickness and spring-back.

The present work proposes a semi-analytical model based on fitting forming force measurements when forming truncated cones for different values of the deformed volume. This deformed volume is determined as a function of the contact surface and thickness. The novelty of this model is to extend the concept of specific energy, used in the orthogonal cutting model to SPIF. In this regard, a new concept called specific forming energy is introduced. This specific forming energy relates the forming forces with the geometric parameters characteristic of the process. The solution is simpler than analytical approaches.

Moreover, a coupled thermo-mechanical numerical model, with a hyperelastic-plastic constitutive equation suitable for polymer sheets, is presented. Unlike other aforementioned models with viscoplastic and viscoelastic constitutive equations, the proposed approach studies a hyperelastic-plastic material, only defined by six material parameters, which considers mechanical and thermal response. The material model parameters are determined minimizing the differences between experimental and simulated material stress-strain curves at different temperatures.

The structure of the paper comprises: Section 2 describes the considered assumptions, and the developed numerical and semi-analytical models; Section 3 explains the setup and tests carried out; Section 4 presents the main results, and discusses the goodness of the proposed approaches with respect to real measurements; and, finally, Section 5 presents the main conclusions.

## 2. Forming Force Models

### 2.1. Semi-Analytical Force Model

A simple method that relates the experimental axial forming forces, with a locally deformed volume, is proposed. Note that the main forming force acts along the *z* direction, so that is the one analyzed. 

Following a similar reasoning such as that used in orthogonal cutting, the local plastic deformation induced during SPIF along the *z* direction requires a power *P*, so that it should be held that:

(1) $$P=F_{z}\cdot \Delta z,$$

where *F_z_* is the axial forming force and *Δz* is the step down.

It stands to reason that the power needed to deform a sheet volume *V* should be constant for a specific material and temperature. This constant *U*, similarly to the machining case in Reference [18], can be called specific forming energy:

(2) $$U=\frac{F_{z}\cdot \Delta z}{V},V=S\cdot t,$$

where *S* is the affected punch-sheet area, and *t* is the mean thickness beneath the punch.

When knowing *S* and *t* at a specific position and *U* for a specific material, it is possible to solve the Equation (2) for obtaining *F*_z_. 

The affected area can be calculated from the expression proposed by Bansal et al. [9] as:

(3) $$S=\frac{\pi D}{4}\left(D/2+\Delta z\right)+\frac{\pi D^{2}}{8\left(\gamma -\alpha \right)}\left(sin\alpha -sin\gamma \right).$$

This area depends of the incremental depth *Δz*, the forming tool diameter *D* and the angle of the sheet *α*, since *γ* can be calculated as:

(4) $$\gamma =arcos\left(1-\frac{2\cdot \Delta z}{D}\right).$$

**Figure 2:**
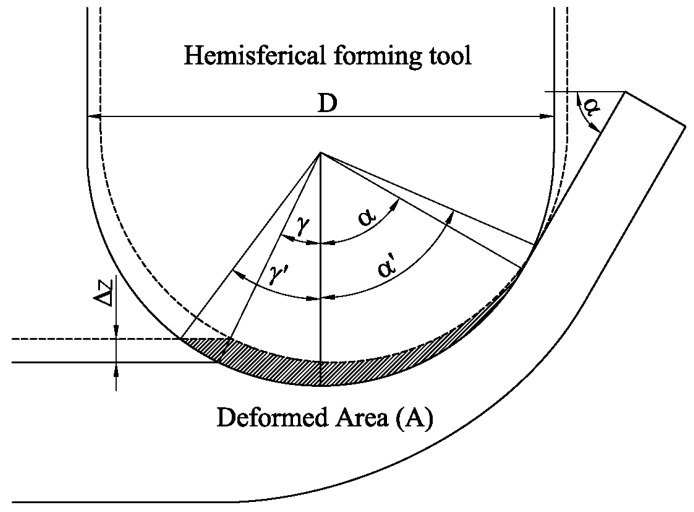
Deformed area defined.

The mean thickness of the deformed volume can be calculated from the sheet area deformed in a step down (Figure 2) and the arc of the punch perimeter in contact with the sheet as:

(5) $$t=\frac{A}{\left(\alpha^{\prime }+\gamma^{\prime }\right)\cdot D/2},$$

(6) $$\alpha^{\prime }=arcos\left(cos\left(\alpha \right)-2\cdot \Delta z/D\right),$$

(7) $$\gamma^{\prime }=arcos\left(1-4\cdot \Delta z/D\right).$$

Based on all of the above, the deformed volume calculated in Equation (2) depends exclusively on the geometric parameters: *Δz*, *D*, and *α*. In summary, once the material characteristic *U* is experimentally determined, the axial forming forces can be estimated from the geometric parameters using Equation (2).

### 2.2. Numerical Estimation of the Forming Force for Polymer Sheets

The numerical model was carried out in a fully coupled-stress dynamic analysis using the ABAQUS commercial software. The analysis considers the inertia effect, the temperature-dependent of the material response, and the transient thermal response. It also includes the integration of the momentum and the heat flow equations coupling the material energy dissipation during plastic flow, rising the local temperature [19].

**Figure 3:**
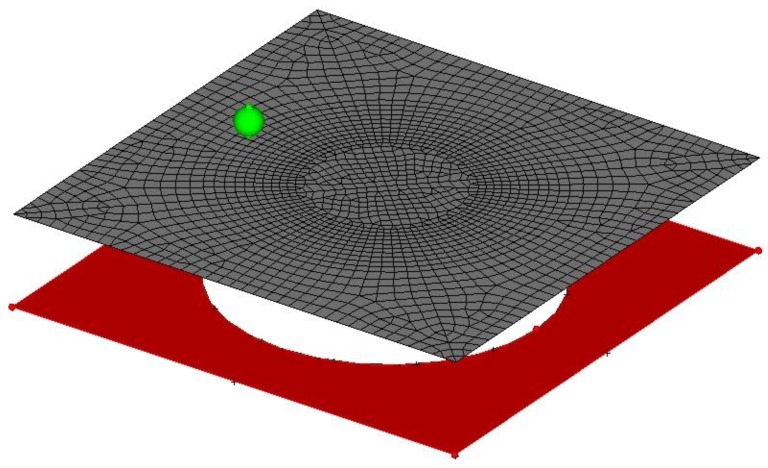
FE model for the SPIF analysis.

The Finite Element (FE) model used for the numerical simulation of the process can be seen in Figure 3. The forming tool and the backing plate were simulated as analytical rigid surfaces in order to perform an efficient numerical contact analysis. The polymer sheet is discretized employing 2052 S4RT elements with reduced integration to avoid the hourglassing, with an active degree of freedom to capture the variation of the temperature at nodes throughout the thickness by bilinear interpolation. The number of integration points used to capture the temperature variation throughout the thickness was 5. The mesh was generated taking into account the tool path that defines the final shape of the part. It was generated uniformly through angle and radius coordinates in order to maintain elements aligned and to generate minimal distortion during the polymer sheet deformation and thickness reduction. The minimum element length in the model was 3 mm, obtaining a minimum stable time increment of 1.2 ×10^−6^ s that is small enough to avoid instabilities during the entire simulation. Note that, mesh size influences the approach accuracy, nevertheless the authors selected the element size in order to limit the computational time to 10 h (for an Intel Core i8 CPU) maintaining an adequate concordance with the experimental results. The contact between the plastic shell and the forming tool was simulated by surface-to-surface interaction with penalty tangential behavior and hard contact behavior for normal direction.

The thermal contact conductance *Ψ* between the metal forming tool and the plastic shell was simulated by gap conductance independent of the contact pressure for polycarbonate (PC) and polyvinyl chloride (PVC) with value of 0.183 W/mK [20]. Other heat transfer mechanisms such as convection or radiation were not taken into consideration due to the low temperature of the plastic shell reached during the entire process. The role of the friction in the formability of the thermoplastics and metals during the SPIF process has been widely discussed by several authors [21,22,23,24,25], concluding that the effect of the friction in the thermoplastic has an important role due to the increase of the temperature of the material. In this work, the values of the dynamic coefficient of friction for the lubricated PC-Shell interface *μ_k_* for PC and PVC are taken from Ludena and Bayer [26]. Thermal properties like conductivity *k_t_* and specific heat *C_p_* are taken from the manufacturer’s data sheet. Table 1 summarized all thermal properties employed in the numerical model.

The material model employed for the two thermoplastics is a non-linear hyperelastic model combined with J_2_-plasticity theory based on isotropic hardening: A simple hardening law that obtains good results with glassy polymers and low material parameters [27]. The hyperelastic component is based on the Arruda-Boyce eight chain model [28] that takes a non-linear Langevin chain statistics into account when deriving the strain energy density function. The predicted stress response of the eight-chain model can be written as follows:

(8) $$\sigma =\frac{\mu }{J\bar{\lambda^{*}}}\frac{{ℒ}^{-1}\left(\bar{\lambda^{*}}/\lambda_{L}\right)}{{ℒ}^{-1}\left(1/\lambda_{L}\right)}\mathrm{dev}\left[b^{*}\right]+\kappa \left(J-1\right)I,$$

where *μ* is the shear modulus, *k* the bulk modulus, and *λ* is the limiting chain stretch. The variable $b^{*}=J^{-2/3}b$ is the distortional left Cauchy-Green tensor, and $\bar{\lambda^{*}}$ is the applied chain stretch which can be calculated from:

(9) $$\bar{\lambda^{*}}=\sqrt{\frac{tr\left[b^{*}\right]}{3}}.$$

The J_2_-plasticity component is based on isotropic hardening, that describes the size change of the yield surface *σ*^0^ as a function of the equivalent plastic strain ${\overset{´}{\varepsilon }}^{pl}$. The model incorporates this effect by an exponential law defined by Equation (10):

(10) $$\sigma^{0}={\sigma |}_{0}+Q_{\infty }\left(1-e^{-b{\overset{´}{\varepsilon }}^{pl}}\right),$$

with, ${\sigma |}_{0}$ the yield stress at zero plastic strain, *Q_∞_* the maximum change of the size in the yield surface and *b* defines the rate at which the size of the yield surface changes as plastic strain develops. If the material is rate independent, the yield condition is:

(11) $$\sigma^{0}=q,$$

with

(12) $$q=\sqrt{\frac{3}{2}S:S},$$

and *S* is the deviatoric stress. The yield function is only dependent on the temperature and the equivalent plastic strain (${\overset{´}{\varepsilon }}^{pl}$).

The procedure to determinate the values of the needed model parameters (*μ*, *k* and *λ*) is described in Section 3.1.2.

## 3. Materials and Methods

### 3.1. Experimental Setup and Polymer Sheets

#### 3.1.1. Setup

**Figure 4:**
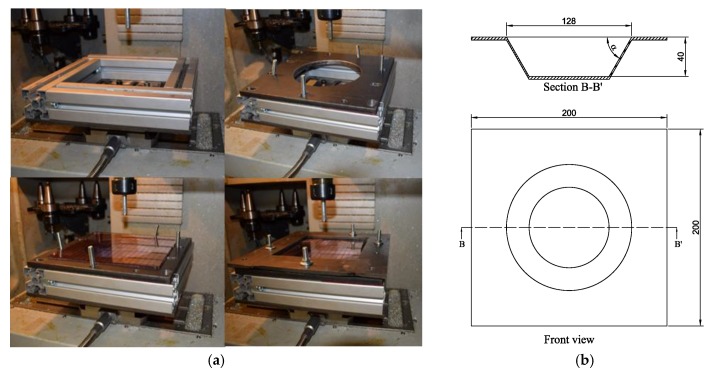
Setup for SPIF: (**a**) CNC machine with fixing system; (**b**) Shape of processed sheet.

The SPIF process is performed using an ALECOP-ODISEA conventional milling machine, with an in-house developed fixing system (Figure 4a). The sheet fixing system is placed on the machining bed, and it consists of a frame made of four aluminum profiles, a die with a hole of 140 mm diameter and an upper die. Eight screws are used to fasten the two dies. Two different tools made of aluminum, with a hemispherical tip of 10 and 12 mm in diameter are used. A lubricant fluid has been used during the experiments.

A 9257BA Kistler dynamometer table is used to measure the value of the force. The dynamometer table is placed between the milling table and the fixing system.

A Flir T335 thermal imaging camera, with a 320 × 240 pixel resolution, is used to measure the temperature reached in the polymer sheet during the process.

Two 200 × 200 mm PVC and PC sheets with a thickness of 3 mm are used. The maximum size of the sheet is the same than that of the working space of the CNC machine to properly fix the sheet. The final shape of the formed sheet is expected to have a 40 mm deep cone, an outer diameter of 128 mm and a cone opening angle *α* with values between 45° and 60°. Figure 4b shows the shape of the specimen and its theoretical dimensions.

#### 3.1.2. Material Properties

**Figure 5:**
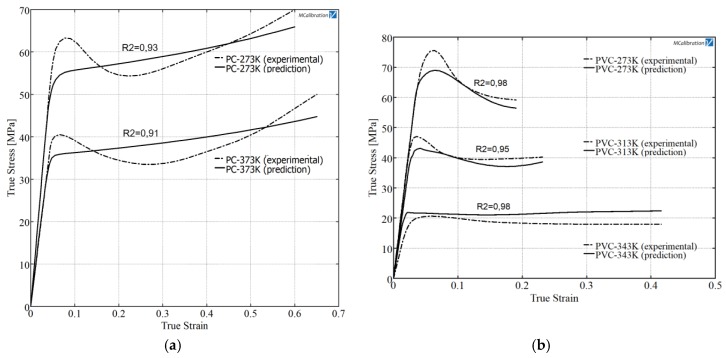
Experimental behavior and calibrated model results at different temperatures for PC (**a**) and PVC (**b**).

The monotonic stress-strain behavior of both glassy polymers, PVC and PC, at different temperatures were characterized by universal testing machine with 6 kN load cell to calibrate the constitutive model, taking into account the temperature material dependence during the simulation. The tests were carried out in uniaxial tension with three replications of each test by following the ASTM D 638-02a norm. The tests were performed at constant strain rate of 1000 mm/min at 273 and 373 K for PC specimens and 273, 313 and 343 K for PVC specimens. It was noticed that both materials experienced a linear elastic response followed by a yielding, after this point the material undergoes softening behavior (Figure 5). The parameters used for the above described material model (Table 2) were calibrated by Mcalibration^®^ commercial software. Instead of an exponential law, a stress-strain tabular data is used to define the evolution of the yield surface size σ^0^. In order to fit PVC and PC material parameters at different temperatures, an optimization method based on Nelder-Mead algorithm was employed. The fitness function was the coefficient of determination *R*^2^, Figure 5 shows the coefficient *R*^2^ for all curves. The calibration was performed with rate independence, for this reason only yield stress values and equivalent plastic strain are provided.

**Figure 6:**
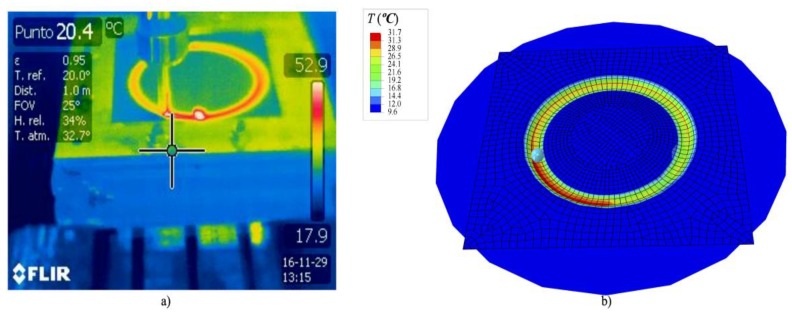
Temperature for a PVC sheet: (**a**) Example of experimental temperature measurements; (**b**) numerical temperature estimation.

In this work, the experimental tests were conducted with lubricant fluid and, therefore, a low temperature variation was noted. Although it is not the aim of this work, once the numerical simulation is validated, it could be used to predict the temperature evolution since the material model was fitted for different temperatures. Figure 6 shows an example of temperature prediction, the gradient of temperature localized in the plastic sheet along the wake generated by the steel tip was due to the dissipated energy converted into heat due to the frictional sliding. This energy is responsible of the increase of temperature in the thermoplastic sheet that requires a temperature-dependent material model to predict the axial force relaxation.

### 3.2. Experimental Procedures

One SPIF test for tuning the numerical model was performed. On the other hand, three tests, changing the deformed volume by means of process parameters, were carried out to determine the semi-analytical approximation. These tests are repeated for each material considered (PVC and PC). In order to validate the models, two additional tests with different conditions to that of the aforementioned tests were carried out.

## 4. Results and Discussion

### 4.1. Tuning Tests

**Figure 7:**
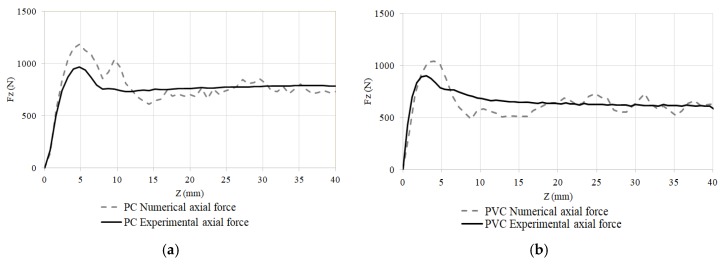
Numerical and experimental axial force profile. (**a**) PC process conditions: Cone shape, *α* = 60°, *D* = 12 mm and *Δz* = 0.8 mm; (**b**) PVC process conditions: Cone shape, *α* = 60°, *D* = 10 mm and *Δz* = 0.6 mm.

The measured axial forming force profile and the numerical estimation, for the tuning tests described in Table 3, are shown in Figure 7. There is an initial transition zone where the forming force grows and a stationary region (*z* > 15 mm) where the force stands approximately constant. The numerical model was fitted to reproduce the stationary region, and the final numerical result is portrayed in Figure 7. For each *z*, the numerical force is the mean of three values obtained at random punch positions, which can be the reason for the oscillation observed in the estimated curve.

### 4.2. Specific Energy Equations

**Figure 8:**
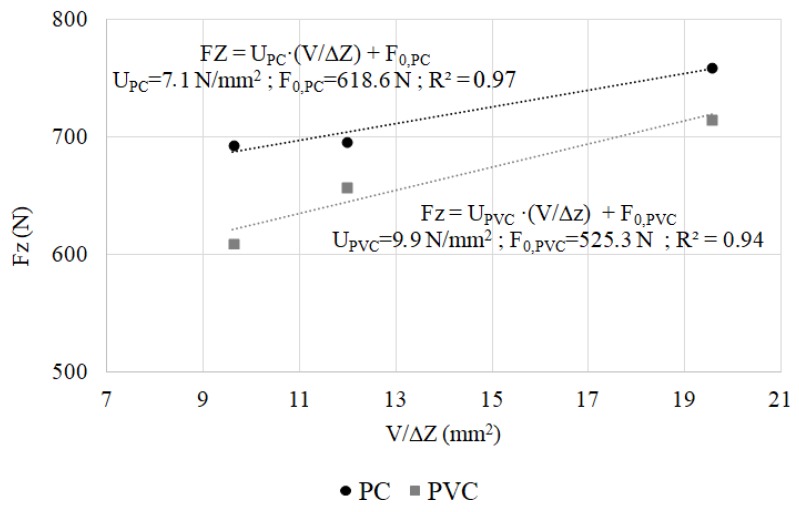
Axial forming forces approximations for PC and PVC.

Figure 8 shows the forces obtained by the semi-analytical model tests described in Table 3. These forces are the mean value of the axial forming force in the stationary region of each of the experimental tests. Except for the intercept, the experimental measurements agree with Equation (2). The intercept can be interpreted as a minimum forming force to obtain a local deformation.

By fitting the experimental data using a linear function, an equation can be obtained that predicts the value of the axial force as a function of the deformed volume and the step down:

(13) $$Fz=U\cdot \frac{V}{\Delta z}+F_{0}$$

where *U* is the specific forming energy and *F*_0_ is the minimum forming force. For each of the tested polymers, different values of the specific forming force and the minimum force were obtained.

The high value of the determination coefficient shows a good agreement between experimental measurements and the linear approximation. 

Only three experimental measurements are enough to obtain the characteristic curve for a specific material. With this curve, any other contact conditions for a material can be computed, predicting the axial forming forces in a simple and accurate way.

### 4.3. Additional Validation Tests

**Figure 9:**
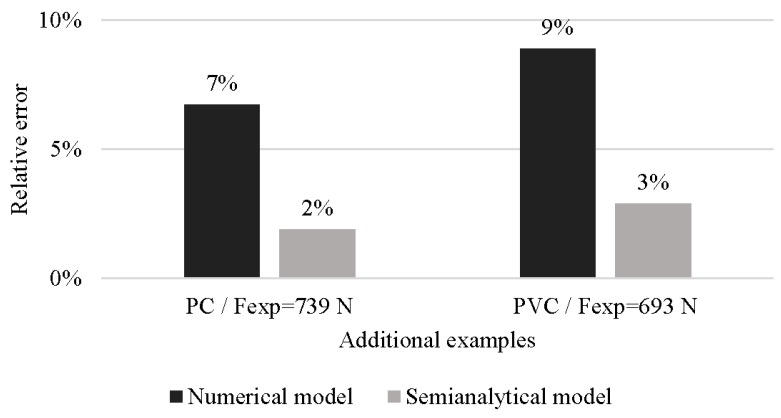
Relative error of the estimation for additional tests. Process conditions: Cone shape, *α* = 60°, *D* = 10 mm and *Δz* = 0.8 mm.

In order to validate the numerical and semi-analytical models, a test using a different condition to the previous tests was carried out. Figure 9 shows the relative error of the estimation models respect to the stationary axial forming force measurements. The two estimation models agree with the actual forces, and have a relative error below 10%. The best result obtained with the semi-analytical model is due to the deformed volume (in this test) is within the fitted region.

**Figure 10:**
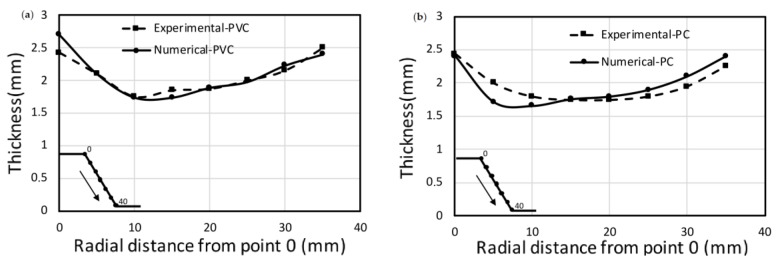
Thickness evolution respect to radial distance, (**a**) PVC and (**b**) PC. Process conditions: Cone shape, *α* = 60°, *D* = 10 mm and *Δz* = 0.8 mm.

The numerical model also provides wall thickness reduction respect to the radial distance (Figure 10). Numerical and experimental thickness measurements are similar for both PVC and PC sheets. The percentage of the mean relative error is always lower than 6% in both cases.

Minimum thickness values are correctly predicted for both polymers. For the PVC sheet, the numerical model also estimates the radial distance where the minimum thickness occurs (maximum stretching). SPIF process uniformly pushes the same percentage of material to the strain direction. At both wall ends, the thickness is greater than that at the initial component wall: Initial thickness is reduced through the wall by stretching the material to the bottom of the component.

## 5. Conclusions

The study of forming force in SPIF is key to improve the process. Poor dimensional accuracy is an important SPIF drawback that can be treated knowing the forming force. There are several analytical, experimental and numerical approaches to determine the forming force but, in general, the existing solutions are focused on metals. For polymers, which also have interesting potential applications, it is difficult to accomplish forming force predictions because of their more complex material behavior.

This work shows two axial forming forces approaches: (1) A simple method based on experimental measurements for different deformation volumes and materials, which extends the specific energy concept used in cutting models to incremental forming processes; and (2) a numerical method, that implements a hyperelastic-plastic material.

Material property curves and the axial forming force at steady state for a set of process conditions were used to tuning the numerical model. Regarding to the semi-analytical solution, we conducted experiments with different axial depth, tool diameter and forming angle in order to study the deformation volume influence on the forming force. These tests were repeated for two polymers PVC and PC.

Once the numerical and the semi-analytical models were ready, we compared their estimations with the real measurements of additional forming tests. The results agree with the experimental measurements, a percentage of relative error below 10% was obtained. 

Extending the semi-analytical procedure to more materials, and further works on this topic could lead to a fast and robust forming force estimation model. On the other hand, the numerical model not only obtained a good forming force prediction, but also additional variables such as thickness evolution estimation, which together with forming force are two of the main variables in any mechanical forming process.

The two approaches could help to improve the knowledge of the SPIF process on polymers. Each solution addresses a research need: Fast computation (semi-analytical model), or low experimental cost (numerical model).

## Figures and Tables

**Table 1:** Thermal properties for polycarbonate (PC) and polyvinyl chloride (PVC) employed in the numerical model.

Property	PC	PVC
Dynamic friction coefficient, *μ_k_*	0.08	0.12
Thermal contact conductance (W/mK), *Ψ*	0.183	0.183
Specific heat (kJ/kgK), *C_p_*	1.25	1.18
Thermal conductance (W/mK), *k_t_*	0.2	0.175
Thermal expansion coefficient (m/mK), *Β*	6.5 × 10^−5^	7 × 10^−5^

**Table 2:** Parameters of the material model proposed.

Material	Temperature	Eight Chain Model Components			Isotropic Hardening		
	(K)	*μ* (MPa)	K	*λ* (MPa)^−1^	*σ_ys_* (MPa)	*σ_ult_* (MPa)	*ε_ult_*
PC	273	805.3	0.0085	2.06	50.2	65.7	0.61
	373	5398.3	0.02	2.36	34.4	42.1	0.66
PVC	273	630.6	0.006	6.65	64.2	58.1	0.18
	313	1040.5	0.0065	4.18	38.1	39.2	0.24
	343	1480	0.0345	8.2	18.2	22.6	0.42

**Table 3:** Experimental tests conducted to define the semi-analytical and numerical model.

Test	Material	*α*	*Δz*	*D*
Tuning tests	PC	60	0.8	12
	PVC	60	0.6	10
Semi-analytical tests	PC & PVC	60	0.5	10
		45	0.5	10
		60	0.8	12
Validation tests	PC & PVC	60	0.8	10
